# Expression of Concern: Facemask wearing to prevent COVID-19 transmission and associated factors among taxi drivers in Dessie City and Kombolcha Town, Ethiopia

**DOI:** 10.1371/journal.pone.0327755

**Published:** 2025-07-08

**Authors:** The PLOS One Editors

The *PLOS One* Editors issue an Expression of Concern to alert readers that this article [1] was identified as one of a series of submissions for which we have concerns about authorship and adherence to the journal’s first and second publication criteria. In addition, the article [1] appears to report the same study and results as an article published later by the same author group [2].

We regret that these issues were not addressed prior to the article’s publication.
