# Supplementary material for: Facemask wearing to prevent COVID-19 transmission and associated factors among taxi drivers in Dessie City and Kombolcha Town, Ethiopia
Source: PLoS One. 2021 Mar 12;16(3):e0247954. doi: 10.1371/journal.pone.0247954 (PMC7954338; doi:10.1371/journal.pone.0247954)
Supplement: S1 Questionnaire — Survey of facemask wearing to prevent COVID-19 transmission and associated factors among taxi drivers in Dessie City and Kombolcha Town, Ethiopia. (DOCX) [file pone.0247954.s001.docx]

Name of interviewer _______________Signature ___________Date __________

Questionnaire ID_________________ Date of data collection_______________

Name of the City/Town ______________ Name of the fermata__________

Data collector name ____________________Signature_________________

Supervisor name ______________________ Signature_________________

Completed/uncompleted questionnaires Completed ______ Uncompleted _______

**Part 1(100) -: Socio- demographic characteristics**

| Code | **Questions** | **Response** |
| --- | --- | --- |
| 101 | Sex | 1. Male B. Female |
| 102 | Age (in years) | ____________ |
| 103 | Educational level | ______________ |
| 104 | Marital status | 1. Single C. Divorced 2. Married D. Widowed |
| 106 | Ethnicity | 1. Amhara C. Tigre 2. Oromo D. Afar   E. Other /specify ____________ |
| 107 | Place of residence | A. Urban B. Rural |
| 108 | Monthly income | _______________ |
| 109 | Household size | _______________ |

**Part II (200):-Questions developed to assess the knowledge of the respondents about COVID-19**

| Code | **Questions** | **Response** | |
| --- | --- | --- | --- |
|  |  | Yes | No |
| 201 | The main clinical symptoms of COVID-19 are fever, fatigue, dry cough, and myalgia |  |  |
| 202 | Stuffy nose, runny nose, and sneezing are less common in persons infected with COVID-19 |  |  |
| 203 | Not all persons with COVID-19 will develop severe cases. Only those who are elderly and with chronic illnesses are more likely to have severe case |  |  |
| 204 | Currently there is no effective cure for COVID-19, but early symptomatic and supportive treatment can help most patients recover from the infection |  |  |
| 205 | The COVID-19 virus spreads via respiratory droplets of infected individuals |  |  |
| 206 | Proper hand washing with soap and water is one method of preventing COVID-19 |  |  |
| 207 | One way to prevent COVID19 is avoiding touching the eye, nose with unwashed hands |  |  |
| 208 | To prevent infection of COVID-19, individuals should avoid going to crowded places such as trains stations and avoid taking public transportation |  |  |
| 209 | People who have contact with someone infected with the COVID-19 virus should be immediately isolated in a proper place |  |  |
| 210 | Isolation and treatment of people who are infected with the COVID-19 virus are effective ways to reduce the spread of the virus |  |  |
| 211 | Children and young adults don’t need to take measures to prevent the infection by the COVID-19 virus |  |  |

**Part III (300):- Questions developed to assess the attitude of the respondents towards COVID-19**

| Code | **Variables** | **Strongly Agree** | **Agree** | **Neutral** | **Disagree** | **Strongly Disagree** |
| --- | --- | --- | --- | --- | --- | --- |
| 301 | When you meet your friends and  colleagues, do you always greet  them with a handshake? |  |  |  |  |  |
| 302 | When you meet your friends and  colleagues, do you always greet  them with a hug? |  |  |  |  |  |
| 303 | Do you wash your hands regularly  and for sufficient length of time? |  |  |  |  |  |
| 304 | Do you usually put on a face mask to  protect yourself from the risk of  Infection? |  |  |  |  |  |
| 305 | If you find that you have contacted  person infected with the virus, do you  inform the health authorities? |  |  |  |  |  |
| 306 | If you have any of the symptoms  associated with the disease, do you  inform the health authorities? |  |  |  |  |  |
| 307 | If you find that you have contacted a  person infected with the virus, do you  agree to be isolated at home for a  certain period of time until it is proven  that you are free from the disease? |  |  |  |  |  |
| 308 | If you are asked to be isolated for a  certain period of time, Do you think  your income should be continued  during this period? |  |  |  |  |  |
| 309 | If there is an available lab test for  detection of the virus, are you willing to be tested? |  |  |  |  |  |
| 310 | If there is an available vaccine for the  virus, are you willing to get it? |  |  |  |  |  |
| 311 | Do you usually follow the updates  about the spread of the virus in your  country? |  |  |  |  |  |
| 312 | Do you usually follow the updates  about the spread of the virus  worldwide? |  |  |  |  |  |
| 313 | If a lecture about the virus is  organized near you, would you be  willing to attend it? |  |  |  |  |  |
| 314 | If flyers or brochures that include  information about the disease are  distributed, would you be willing to  read them and follow the instructions  mentioned in them? |  |  |  |  |  |
| 315 | If protective measures and equipment  are available at an affordable price,  would you be willing to buy them? |  |  |  |  |  |

**Part IV(400): Questions related with face mask wearing**

| Code | **Questions** | **Response** |
| --- | --- | --- |
| 401 | Does the driver wear a facemask? (Observational) | A. Yes B. No |
| 402 | How often do you wear a facemask? | A. Always  B. Sometimes  C. Never |
| 403 | What type of facemask does he/she use? (Observational) | A. Surgical/medical facemasks  B. Cloth facemasks  C. N95 facemask  D. Other (Specify) _________ |
| 404 | Which part of the face does the worn facemask cover? (Observational) | A. Nose and mouth  B. Nose, mouth, lower jaw and facial hair  C. Other (Specify) ________ |
| 405 | For how long do you wear a facemask? | ___________________ |
| 406 | How do you keep the cleanliness of the face mask? (only for reusable face masks) | 1. Washing with water 2. Washing with hot water and soap and drying in hot sun for 5 hours 3. Washing and boiling for 5 minutes and drying 4. Ironing the mask for 5 minutes daily 5. Other (Specify)___________ |
| 407 | Where do you dispose the face mask? | A. Open disposal  B. Pit  C. Burning  D. Other (Specify)___________ |

**Part V (500): Questions related with behavioural factors affecting face mask wearing**

| Code | **Questions** | **Response** |
| --- | --- | --- |
| 501 | Do you feel as you are vulnerable to contracting COVID-19? | A. Yes B. No |
| 502 | Do you know any individuals infected with COVID-19? | A. Yes B. No |
| 503 | Do you fear of COVID-19? | A. Yes B. No |
| 504 | Do you worry that Dessie City/Kombolcha Town would become a quarantine City/Town because of the widespread of COVID-19 to the community? | A. Yes B. No |
| 505 | Do you believe that wearing a face mask can be effective in preventing contracting and spreading of COVID-19? | A. Yes B. No |
| 506 | Do you face difficulty in obtaining face mask? | A. Yes B. No |
| 507 | Do you face discomfort when wearing a face mask? | A. Yes B. No |
| 508 | Is there a government pressure to wear a face mask? | A. Yes B. No |
| 509 | Do your family members encourage you to wear face mask? | A. Yes B. No |
| 510 | Do passengers encourage you to wear face mask? | A. Yes B. No |
| 511 | Is wearing face mask is acceptable in your working area? | A. Yes B. No |
| 512 | Do you know as COVID-19 can be a fatal disease? | A. Yes B. No |

Thank you
