# Supplementary material for: Facemask wearing to prevent COVID-19 transmission and associated factors among taxi drivers in Dessie City and Kombolcha Town, Ethiopia
Source: PLoS One. 2021 Mar 12;16(3):e0247954. doi: 10.1371/journal.pone.0247954 (PMC7954338; doi:10.1371/journal.pone.0247954)
Supplement: S2 Questionnaire — Survey of facemask wearing to prevent COVID-19 transmission and associated factors among taxi drivers in Dessie City and Kombolcha Town, Ethiopia. (DOCX) [file pone.0247954.s002.docx]

የመጠይቁ ኮድ_______________ቀን_______________

የከተማዉ ስም ________________የፌርማታዉ ስም _____________

የመረጃ ሰብሳቢው ስም_________________ ፊርማ__________________

የተቆጣጣሪው ስም ___________________ ፊርማ___________

የተሟላ መጠይቅ /ያልተሟላ መጠይቅ የተሟላ________ ያልተሟላ _______

**ክፍል አንድ(100)፡ ማህበራዊና ኢኮኖሚያዊ ሁኔታዎች**

| **ኮድ** | **መጠይቅ** | መልሶች |
| --- | --- | --- |
| 101 | የመረጃ ሰጪው ፆታ | 1. ወንድ **2.** ሴት |
| 102 | የመረጃ ሰጪው ዕድሜ **(**በዓመት) | ____________ |
| 103 | የትምህርት ሁኔታ | ______________ |
| 104 | የጋብቻ ሁኔታ | 1. ያላገባ  2. ያገባ  3. ሚስቱ በሞት የተለየ**ች**በት  4. የፈታ |
| 105 | ሀይማኖት | _____________ |
| 106 | ብሄር | ______________ |
| 107 | የመኖርያ አድራሻ | 1. ከተማ 2. ገጠር |
| 108 | የቤተሰቡ ወርሃዊ የገቢ መጠን | _______________ |
| 109 | የቤተሰብ ብዛት | _______________ |

**ክፍል ሁለት (200):- የጥናቱ ተሳታፊዎችን የኮሮና ቫይረስ የዕዉቀት ደረጃ ለመለካት የተዘጋጀ መጠይቅ**

| **ኮድ** | **መጠይቅ** | መልሶች |
| --- | --- | --- |
| 201 | የኮሮና ቫይረስ ዋነኛ ምልክቶች ትኩሳት፤ መዛል ፤ደረቅ ሳልና የጡንቻ ህመም ናቸዉ፡፡ | 0. የለም 1. አዎ |
| 202 | ከጉንፋን በተቃራኒ የአፍንጫ ፈሳሽ፤ የአፍንጫ መዝጋትና ማስነጠስ በኮሮና ቫይረስ በተያዘ ሰዉ ላይ እምብዛም/ብዙ የተለመዱ አይደሉም፡፡ | 0. የለም 1. አዎ |
| 203 | ሁሉም በኮሮና ቫይረስ የተያዙ ሰዎች የጠና ህመም አይታይባቸዉም፡፡ ነገር ግን በእድሜ ገፋ ያሉና ተጉዳኝ በሽታዎች ያሉባቸዉ ስዎች በሽታዉ ሊጠናባቸዉ ይችላል ፡፡ | 0. የለም 1. አዎ |
| 204 | በአሁን ጊዜ ለኮሮና ቫይረስ መድሃኒት የሌለዉ ሲሆን ምልክቶች መታየት ሲጀምሩ በጊዜ የሚደረጉ የህክምና ዕርዳታዎች ታማሚዎች እንዲያገግሙ ያደርጋል ፡፡ | 0. የለም 1. አዎ |
| 205 | የኮሮና ቫይረስ የሚሰራጨዉ በበሽታዉ የተያዘ ሰዉ በሚተነፍስባት ጊዜ በሚወጡ ረቂቅ ተዋስያን ነዉ፡፡ | 0. የለም 1. አዎ |
| 206 | እጅን በሳሙናና በዉሃ በአግባቡ መታጠብ በሽታዉን ለመከላከል ሁነኛ መንገድ ነዉ፡፡ | 0. የለም 1. አዎ |
| 207 | አይንን ፤አፍንጫን እና አፍን ባልታጠበ እጅ ባለመንካት የቫይረሱን ስርጭት መግታት እንችላለን ፡፡ | 0. የለም 1. አዎ |
| 208 | የተጨናነቁና ሕዝብ የሚበዛባቸዉ ቦታዎች ባለመሄድ የቫይረሱን ስርጭት መግታት ይቻላል፡፡ | 0. የለም 1. አዎ |
| 209 | በቫይረሱ መያዙ የተረጋገጠ ሰዉ በአፋጣኝ ራሱን ለይቶ ማቆየት አለበት ፡፡ | 0. የለም 1. አዎ |
| 210 | በቫይረሱ የተያዙ ሰዎች ላይ የሚደረገዉ ለይቶ ማቆየትና ሕክምና የቫይረሱን ስርጭት ለመግታት ይረዳል ፡፡ | 0. የለም 1. አዎ |
| 211 | ልጆችና ወጣቶች በቫይረሱ ላለመያዝ ጥንቃቄ ማድረግ አይጠበቅባቸዉም፡፡ | 0. የለም 1. አዎ |

**ክፍል 3(300) ፡- የጥናቱ ተሳታፊዎችን የኮሮና ቫይረስ የዕይታ ደረጃ ለመለካት የተዘጋጀ መጠይቅ**

| **ኮድ** | **መጠይቅ** | መልሶች |
| --- | --- | --- |
| 301 | ጉደኞችህን እና ባልደረቦችህን በምታገኝበት ጊዜ ሁሉ ሰላምታ የምትለዋወጠዉ እጅ በመጨባበጥ ነዉ? | 1. በጣም እስማማለሁ  2. እስማማለሁ  3. ገለልተኛ  4. አልስማማም  5. በጣም አልስማማም |
| 302 | ጉዳኞችህን እና ባልደረቦችህን በምታገኝበት ጊዜ ሁሉ ሰላምታ የምትለዋወጠዉ በመተቃቀፍ ነዉ? | 1. በጣም እስማማለሁ  2. እስማማለሁ  3. ገለልተኛ  4. አልስማማም  5. በጣም አልስማማም |
| 303 | እጆችህን ዘወትር በዉሃና በሳሙና ቢያንስ ለ20 ሰከንድ ያህል ትታጠባለህ? | 1. በጣም እስማማለሁ  2. እስማማለሁ  3. ገለልተኛ  4. አልስማማም  5. በጣም አልስማማም |
| 304 | የቫይረሱን ስርጭት ለመከላከል አብዛኛዉን ጊዜ የአፍና አፍንጫ መሸፊኛ ጭምብል ታደርጋለህ? | 1. በጣም እስማማለሁ  2. እስማማለሁ  3. ገለልተኛ  4. አልስማማም  5**.** በጣም አልስማማም |
| 305 | በቫይረሱ ከተያዘዉ ሰዉ ጋር ግንኙነት እንዳለህ ካረጋገጥክ ለጤና ባለሞያዎች ታሳዉቃለህ? | 1. በጣም እስማማለሁ  2. እስማማለሁ  3. ገለልተኛ  4. አልስማማም  5. በጣም አልስማማም |
| 306 | የበሽታዉ ምልክቶች ከታዩብህ ለጤና ባለሞያዎች ታሳዉቃለህ? | 1**.** በጣም እስማማለሁ  **2.** እስማማለሁ  **3.** ገለልተኛ  4**.** አልስማማም  **5.** በጣም አልስማማም |
| 307 | በበሽታዉ ከተያዘ ሰዉ ጋር ግንኙነት እንዳለህ ካረጋገጥክ በበሽታዉ መያዝህ እስኪረጋገጥ እራስህን እቤት ዉስጥ በማግለል ለማቆየት ትስማማለህ? | 1**.** በጣም እስማማለሁ  2. እስማማለሁ  3. ገለልተኛ  4**.** አልስማማም  5**.** በጣም አልስማማም |
| 308 | ለተወሰነ ጊዜ ራስህን እንድታገልል ከተጠየክ የማገኘዉ ገቢ መቀጠል አለበት ብለህ ታስባለህ? | 1. በጣም እስማማለሁ  2. እስማማለሁ  3. ገለልተኛ  4. አልስማማም  5**.** በጣም አልስማማም |
| 309 | ቫይረሱን ለመለየት የሚደረግ ላቦራቶሪ ምርመራ ካለ ለማድረግ ዝግጁ ነህ? | 1. በጣም እስማማለሁ  2. እስማማለሁ  3. ገለልተኛ  4. አልስማማም  5. በጣም አልስማማም |
| 310 | ለበሽታዉ የሚሆን መድሃኒት ካለ ለመቀበል ዝግጁ ነህ? | 1. በጣም እስማማለሁ  2. እስማማለሁ  3. ገለልተኛ  4. አልስማማም  5. በጣም አልስማማም |
| 311 | ስለበሽታዉ በየጊዜዉ የሚሰጡ ሀገራዊ መረጃዎችን ትከታተላለህ? | 1. በጣም እስማማለሁ 2.እስማማለሁ  3. ገለልተኛ  4. አልስማማም  5**.** በጣም አልስማማም |
| 312 | ስለበሽታዉ በየጊዜዉ የሚሰጡ አለማቀፋዊ መረጃዎችን ትከታተላለህ? | 1. በጣም እስማማለሁ  2. እስማማለሁ  3. ገለልተኛ  4. አልስማማም  5. በጣም አልስማማም |
| 313 | በሽታዉን በተመለከተ የሚዘጋጁ ትምህርታዊ ዝግጅቶችን ትከታተላለህ? | 1. በጣም እስማማለሁ  2. እስማማለሁ  3. ገለልተኛ  4. አልስማማም  5. በጣም አልስማማም |
| 314 | ስለበሽታዉ መረጃ የያዙ በራሪ ወረቀቶችን ስታገኝ ታነባለህ መመርያዎቹንም ትከተላለህ? | 1. በጣም እስማማለሁ  2. እስማማለሁ  3. ገለልተኛ  4. አልስማማም  5. በጣም አልስማማም |
| 315 | በሸታዉን ለመቆጣጠር የሚሆኑ መሳርያዎች በተመጣጣኝ ዋጋ ከተገኘ ትገዛለህ? | 1. በጣም እስማማለሁ  2. እስማማለሁ  3. ገለልተኛ  4. አልስማማም  5**.** በጣም አልስማማም |

**ክፍል 4(400) ፡- ከአፍና አፍንጫ መሸፊኛ** **ጭምብል አጠቃቀም ጋር የተያያዙ ጥያቄዎች**

| **ኮድ** | **መጠይቅ** | መልሶች |  |
| --- | --- | --- | --- |
| 401 | ሹፌሩ የአፍና አፍንጫ መሸፊኛ ጭምብል አድርጓል? (በምልከታ የሚመለስ) | 0. የለም 1. አዎ |  |
| 402 | የአፍና አፍንጫ መሸፈኛጭምብል መቼ መቼ ታደርጋለህ? | 1. ዘወትር  2. አልፎ አልፎ  3. ጭራሽ | |
| 403 | ሹፌሩ ምን አይነት ጭምብል ነዉ ያደረገዉ? (በምልከታ የሚመለስ) | ሀ) ሰርጂካል  ለ) N95  ሐ) የጨርቅ  መ) ሌላ(ይገለጽ)_____________ |  |
| 404 | ያደረገዉ ጭምብል የትኛዉን የፊት ክፍል ሸፍኗል? (በምልከታ የሚመለስ) | ሀ) አፍንጫና አፍ  ለ) አፍንጫ፤አፍ፤የታችኛዉን አገጭና ጺም  ሐ.ሌላ(ይገለጽ)_______________ |  |
| 405 | ጭምብሉን ለምን ያክል ጊዜ ታደርጋለህ? | ___________________ |  |
| 406 | የጭምብሉን ንጽህና እንዴት ነዉ የምትጠብቀዉ? (መልሶ ለመጠቀም ለሚያገለግል ጭምብል | ሀ) በዉሃ በማጠብ  ለ) በሞቀ ዉሃና በሳሙና በማጠብና ለ5ደቂቃ በፀሀይ በማድረቅ  ሐ) በማጠብና በመቀቀል እና ለ5 ደቂቃ በፀሀይ በማድረቅ  መ) ጭምብሉን ለ5 ደቂቀ በመተኮስ  ሠ)ሌላ(ይገለጽ)_______________ |  |
| 407 | ጭምብሉን የት ነዉ የምታስወግደዉ? (ከአንድ በላይ መመለስ ይቻላል) | ሀ) ሜዳ ላይ በመወርወር  ለ) መኪና ዉስጥ  ሐ)በማቃጠል መ)ሌላ(ይገለጽ)____________ |  |

**ክፍል 5(500) ፡- ከባህሪ ጋር የተያያዙ ሁኔታዎች**

| **ኮድ** | **መጠይቅ** | መልሶች |
| --- | --- | --- |
| 501 | ለኮሮና ቫይረስ ተጋላጭ ነኝ ብለህ ታስባለህ? | 0. የለም 1. አዎ |
| 502 | በኮሮና ቫይረስ የተያዘ ሰዉ ታዉቃለህ? | 0. የለም 1. አዎ |
| 503 | የኮሮና ቫይረስን ትፈራለህ? | 0. የለም 1. አዎ |
| 504 | የደሴ/ኮምቦልቻ ከተማ የለይቶ ማቆያ ማእከል ይሆናል ብለህ ትጨነቃለህ? | 0. የለም 1. አዎ |
| 505 | የአፍና አፍንጫ መሸፊኛ ጭምብል የኮሮና በሽታ ስርጭትንና መተላለፍን ይከላከላል ብለህ ታስባለህ? | 0. የለም 1. አዎ |
| 506 | የአፍና አፍንጫ መሸፊኛ ጭምብል ለማግኘት ተቸግረአል? | 0. የለም 1. አዎ |
| 507 | የአፍና አፍንጫ መሸፊኛ ጭምብል በምትጠቀምበት ጊዜ ያለመመቸት ችግር አለ? | 0. የለም 1. አዎ |
| 508 | መንግስት የአፍና አፍንጫ መሸፊኛ ጭምብል እንድታደርግ ግፊት ያሳድርብሃል? | 0. የለም 1. አዎ |
| 509 | የቤተሳብህ አባላቶች የአፍና አፍንጫ መሸፊኛ ጭምብል እንድታደርግ ያበረታቱሀል? | 0. የለም 1. አዎ |
| 510 | ተሳፋሪወች የአፍና አፍንጫ መሸፊኛ ጭምብል እንድታደርግ ያበረታቱሀል? | 0. የለም 1. አዎ |
| 511 | በስራህ ቦታ ላይ የአፍና አፍንጫ መሸፊኛ ጭምብል መጠቀም ተቀባይነት አለዉ? | 0. የለም 1. አዎ |
| 512 | የኮሮናን ቫይረስ ገዳይ መሆኑን ታዉቃለህ? | 0. የለም 1. አዎ |

**ስለተሳትፎህ በጣም አመሰግናለሁ!!!**
