# Supplementary material for: Facemask wearing to prevent COVID-19 transmission and associated factors among taxi drivers in Dessie City and Kombolcha Town, Ethiopia
Source: PLoS One. 2021 Mar 12;16(3):e0247954. doi: 10.1371/journal.pone.0247954 (PMC7954338; doi:10.1371/journal.pone.0247954)

PLOS ONE

Proportion of ~~facemask~~ wearing to prevent COVID-19 and associated factors among taxi drivers in urban areas of Dessie ~~City~~ and Kombolcha Towns in Ethiopia

--Manuscript Draft--

**Manuscript Number:**	PONE-D-20-33269
**Article Type:**	Research Article
**Full Title:**	Proportion of facemask wearing to prevent COVID-19 and associated factors among taxi drivers in urban areas of Dessie City and Kombolcha Towns in Ethiopia
**Short Title:**	Facemask wearing to prevent COVID-19 and associated factors among taxi drivers
**Corresponding Author:**	Metadel Adane (PhD)<br>Wollo University<br>Dessie, ETHIOPIA
**Keywords:**	COVID-19; Facemask Wearing; Taxi Drivers; Ethiopia

Abstract:

Background

Corona virus infectious disease-19 (COVID-19) is an outbreak which causes morbidity and mortality throughout the world. This disease can be spread in a rapid rate if major control measures are not practiced well. World Health Organization (WHO) pointed out that taxi drivers and users ~~of taxi~~ in urban areas are at higher risk of COVID-19 due to frequent contact among many peoples. Facemask wearing is one of the recommended options for taxi drivers should frequently implement to control the transmission of the virus. However, lack of evidence on the status of facemask wearing and associated factors among taxi drivers in Dessie City and Kombolcha Towns hinders for proper planning and advocacy of facemask use, where this study was designed to address these gaps by providing evidence based result regarding the proportion and associated factors of facemask wearing among taxi drivers in urban areas Dessie City and Kombolcha Towns, Ethiopia.

Methods

A cross-sectional study was conducted among 417 taxi drivers in Dessie City and Kombolcha towns during June to August, 2020. The study participants were selected using simple random sampling technique after proportionally allocated the sample size for the total taxi drivers working in Dessie City and Kombolcha towns. The data was collected by trained data collectors using structured questionnaire and on-spot observational checklist. The collected data was checked, coded and entered to Epi-data version 3.4 and exported to statistical package for social sciences (SPSS) version 25.0 for analysis. Bivariable (Crude Odds Ratio [COR] and multivariable (Adjusted Odds Ratio [AOR]) analysis were employed using logistic regression model with 95% CI (confidence interval). From bivariable analysis variables with $p < 0.25$ was retained in to multivariable analysis. Then, from the multivariable analysis, variables with $p < 0.05$ were considered as factors significantly associated with facemask wearing among taxi drivers in Dessie City and Kombolcah towns.

Results

The proportion of facemask wearing among taxi drivers was 54.68% [95%CI: 50.1 - 59.7] and the proportion who did not wear facemask was 45.32% [95%CI: 40.3 - 49.9]. The majority 243(58.3%) of the drivers were using cloth facemasks, followed by N95 facemasks 102(24.5%) and surgical facemasks 72(17.3%). Out of the total 417 taxi drivers the majority 291(69.8%) of them had a good knowledge about COVID-19 were as some 126(30.2%) had poor knowledge. With regard to attitude about the disease the majority 282(67.6%) had good attitude and some 135(32.4%) had poor attitude towards COVID-19. We found that marital status [AOR=3.14, 95%CI: 1.97-5.01], fear of the disease [AOR=2.1, 95%CI: 1.28-3.47], believing in the effectiveness of facemask [AOR=5.6, 95%CI: 3.1-10.16] and government pressure [AOR=3.6, 95%CI: 2.16-6.13] were factors significantly associated with facemask wearing.

Conclusion

We found that the proportion of facemask wearing among taxi drivers was relatively low in Dessie City and Kombolcha Towns. In order to prevent COVID-19 transmission, we recommended that government and non-government organization should have to work very closely to increase the proportion of facemask wearing by implementing facemask use advocacy and promotion, availability of facemask with low price

Powered by Editorial Manager® and ProduXion Manager® from Aries Systems Corporation

	monitoring and controlling of facemask use.
Order of Authors:	Tarikuwa Natnael
	Metadel Adane (PhD)
	Yeshiwork Alemnew
	Gete Berihun
	Masresha Abebe
	Atsedemariam Andualem
	Sewunet Ademe
	Belachew Tegegne
Additional Information:	
Question	Response
**Financial Disclosure**<br><br>Enter a financial disclosure statement that describes the sources of funding for the work included in this submission. Review the submission guidelines for detailed requirements. View published research articles from *PLOS ONE* for specific examples.<br><br>This statement is required for submission and **will appear in the published article** if the submission is accepted. Please make sure it is accurate.<br><br>**Unfunded studies**<br>Enter: *The author(s) received no specific funding for this work.*<br><br>**Funded studies**<br>Enter a statement with the following details:<br>• Initials of the authors who received each award<br>• Grant numbers awarded to each author<br>• The full name of each funder<br>• URL of each funder website<br>• Did the sponsors or funders play any role in the study design, data collection and analysis, decision to publish, or preparation of the manuscript?<br>• **NO** - Include this sentence at the end of your statement: *The funders had no role in study design, data collection and analysis, decision to publish, or preparation of the manuscript.*<br>• **YES** - Specify the role(s) played.<br><br>* typeset	Wollo University funded this study. The funders had no role in study design, data collection and analysis, decisions to publish, interpretation of the data and preparation of the manuscript for publication.

Powered by Editorial Manager® and ProduXion Manager® from Aries Systems Corporation

**Competing Interests**<br><br>Use the instructions below to enter a competing interest statement for this submission. On behalf of all authors, disclose any competing interests that could be perceived to bias this work—acknowledging all financial support and any other relevant financial or non-financial competing interests.<br><br>This statement **will appear in the published article** if the submission is accepted. Please make sure it is accurate. View published research articles from *PLOS ONE* for specific examples.<br><br>**NO authors have competing interests**<br><br>Enter: *The authors have declared that no competing interests exist.*<br><br>**Authors with competing interests**<br><br>Enter competing interest details beginning with this statement:<br><br>*I have read the journal's policy and the authors of this manuscript have the following competing interests: [insert competing interests here]*<br><br>* typeset	The authors have declared that no competing interests exist.
**Ethics Statement**<br><br>Enter an ethics statement for this submission. This statement is required if the study involved:<br><br>• Human participants<br>• Human specimens or tissue<br>• Vertebrate animals or cephalopods<br>• Vertebrate embryos or tissues<br>• Field research<br><br>Write "N/A" if the submission does not require an ethics statement.<br><br>General guidance is provided below.	Ethical clearance was obtained from the ethical review committee of Wollo University College of Medicine and Health Sciences. Before each interview, data collectors explained the purpose of the study, gave assurance of confidentiality, addressed any other ethical issues and obtained informed verbal consent from each taxi drivers.

Powered by Editorial Manager® and ProduXion Manager® from Aries Systems Corporation

Consult the submission guidelines for detailed instructions. **Make sure that all information entered here is included in the Methods section of the manuscript.**<br><br>**Format for specific study types**<br><br>**Human Subject Research (involving human participants and/or tissue)**<br>• Give the name of the institutional review board or ethics committee that approved the study<br>• Include the approval number and/or a statement indicating approval of this research<br>• Indicate the form of consent obtained (written/oral) or the reason that consent was not obtained (e.g. the data were analyzed anonymously)<br><br>**Animal Research (involving vertebrate animals, embryos or tissues)**<br>• Provide the name of the Institutional Animal Care and Use Committee (IACUC) or other relevant ethics board that reviewed the study protocol, and indicate whether they approved this research or granted a formal waiver of ethical approval<br>• Include an approval number if one was obtained<br>• If the study involved *non-human primates*, add *additional details* about animal welfare and steps taken to ameliorate suffering<br>• If anesthesia, euthanasia, or any kind of animal sacrifice is part of the study, include briefly which substances and/or methods were applied<br><br>**Field Research**<br><br>Include the following details if this study involves the collection of plant, animal, or other materials from a natural setting:<br>• Field permit number<br>• Name of the institution or relevant body that granted permission	
**Data Availability**<br><br>Authors are required to make all data underlying the findings described fully available, without restriction, and from the	Yes - all data are fully available without restriction

Powered by Editorial Manager® and ProduXion Manager® from Aries Systems Corporation

time of publication. PLOS allows rare exceptions to address legal and ethical concerns. See the PLOS Data Policy and FAQ for detailed information.<br><br>A Data Availability Statement describing where the data can be found is required at submission. Your answers to this question constitute the Data Availability Statement and **will be published in the article**, if accepted.<br><br>**Important:** Stating 'data available on request from the author' is not sufficient. If your data are only available upon request, select 'No' for the first question and explain your exceptional situation in the text box.<br><br>Do the authors confirm that all data underlying the findings described in their manuscript are fully available without restriction?	
**Describe where the data may be found in full sentences. If you are copying our sample text, replace any instances of XXX with the appropriate details.**<br><br>• If the data are **held or will be held in a public repository**, include URLs, accession numbers or DOIs. If this information will only be available after acceptance, indicate this by ticking the box below. For example: *All XXX files are available from the XXX database (accession number(s) XXX, XXX.)*.<br>• If the data are all contained **within the manuscript and/or Supporting Information files**, enter the following: *All relevant data are within the manuscript and its Supporting Information files.*<br>• If neither of these applies but you are able to provide **details of access elsewhere**, with or without limitations, please do so. For example:<br><br>*Data cannot be shared publicly because of [XXX]. Data are available from the XXX Institutional Data Access / Ethics Committee (contact via XXX) for*	All relevant data are within the manuscript and its Supporting Information files.

researchers who meet the criteria for access to confidential data.<br><br>*The data underlying the results presented in the study are available from (include the name of the third party and contact information or URL).*<br>• This text is appropriate if the data are owned by a third party and authors do not have permission to share the data.<br><br>* typeset	
Additional data availability information:	

Powered by Editorial Manager® and ProduXion Manager® from Aries Systems Corporation

Manuscript Click here to access/download;Manuscript;Manuscript.docx

Proportion of ~~facemask~~ wearing to prevent COVID-19 and associated factors among taxi drivers in urban areas of Dessie ~~City~~ and Kombolcha Towns in Ethiopia

Tarikuwa Natnael[1#], Metadel Adane [1*#], Yeshiwork Alemnew[2], Gete Berihun[1], Masresha Abebe[1], Atsedemariam Andualem[3], Sewunet Ademe[3], Belachew Tegegne[3]

[1]Department of Environmental Health, College of Medicine and Health Sciences, Wollo University, Dessie, Ethiopia

[2]Department of Biology, College of Natural and Computational Sciences, Wollo University, Dessie, Ethiopia

[3]Department of Nursing, College of Medicine and Health Sciences, Wollo University, Dessie, Ethiopia

*Corresponding author: metadel.adane2@gmail.com

[#]those authors contributed equally

Abstract

Background

Corona virus infectious disease-19 (COVID-19) is an outbreak which causes morbidity and mortality throughout the world. This disease can be spread in a rapid rate if major control measures are not practiced well. World Health Organization (WHO) pointed out that taxi drivers and users of taxi in urban areas are at higher risk of COVID-19 due to frequent contact among many peoples. Facemask wearing is one of the recommended options for taxi drivers should frequently implement to control the transmission of the virus. However, lack of evidence on the status of facemask wearing and associated factors among taxi drivers in Dessie City and Kombolcha Towns hinders for proper planning and advocacy of facemask use, where this study was designed to address these gaps by providing evidence based result regarding the proportion and associated factors of facemask wearing among taxi drivers in urban areas Dessie City and Kombolcha Towns, Ethiopia.

Methods

A cross-sectional study was conducted among 417 taxi drivers in Dessie City and Kombolcha towns during June to August, 2020. The study participants were selected using simple random sampling technique after proportionally allocated the sample size for the total taxi drivers working in Dessie City and Kombolcha towns. The data was collected by trained data collectors using structured questionnaire and on-spot observational checklist. The collected data was checked, coded and entered to Epi-data version 3.4 and exported to statistical package for social sciences (SPSS) version 25.0 for analysis. Bivariable (Crude Odds Ratio [COR] and multivariable (Adjusted Odds Ratio [AOR]) analysis were employed using logistic regression model with 95% CI (confidence interval). From bivariable analysis,

Variables with $p < 0.25$ was retained in to multivariable analysis. Then, from the multivariable analysis, variables with $p < 0.05$ were considered as factors significantly associated with facemask wearing among taxi drivers in Dessie City and Kombolcah towns.

Results

The proportion of facemask wearing among taxi drivers was 54.68% [95%CI: 50.1 -59.7] and the proportion who did not wear facemask was 45.32% [95%CI: 40.3 - 49.9]. The majority 243(58.3%) of the drivers were using cloth facemasks, followed by N95 facemasks 102(24.5%) and surgical facemasks 72(17.3%). Out of the total 417 taxi drivers the majority 291(69.8%) of them had a good knowledge about COVID-19 were as some 126(30.2%) had poor knowledge. With regard to attitude about the disease the majority 282(67.6%) had good attitude and some 135(32.4%) had poor attitude towards COVID-19. We found that marital status [AOR=3.14, 95%CI: 1.97-5.01], fear of the disease [AOR=2.1, 95%CI: 1.28-3.47], believing in the effectiveness of facemask [AOR=5.6, 95%CI: 3.1-10.16] and government pressure [AOR=3.6, 95%CI: 2.16-6.13] were factors significantly associated with facemask wearing.

Conclusion

We found that the proportion of facemask wearing among taxi drivers was relatively low in Dessie City and Kombolcha Towns. In order to prevent COVID-19 transmission, we recommended that government and non-government organization should have to work very closely to increase the proportion of facemask wearing by implementing facemask use advocacy and promotion, availability of facemask with low price, monitoring and controlling of facemask use.

Keywords: COVID-19, Facemask Wearing, Taxi Drivers, Ethiopia

Introduction

Severe acute respiratory syndrome coronavirus-2 (SARS-CoV-2), formally named as the novel Coronavirus-2019 (nCoV-2019) by the World Health Organization (WHO) originated in the province of Hubei, China during late December 2019 (Zhu *et al*, 2020). Corona virus infectious disease-19 (COVID-19) outbreak becomes the public health problems of the world (Phelan *et al*. 2020).

The clinical symptoms of COVID-19 are fever, dry cough, breathing difficulties, headache, pneumonia, and myalgia or fatigue (Huang *et al*, 2020; Zhou *et al*, 2020). In addition, severe cases can also lead to cardiac injury, respiratory failure, acute respiratory distress syndrome and ultimately death (Holshue *et al*, 2020)**.** At the beginning, COVID-19 was transmitted from animal-to-human, and then it started human-to-human transmission through respiratory droplets and body contacts (Chan *et al*, 2020; Li *et al*, 2020**)**. Another more recent study also found out that transmission through talking, with louder speech (Anfinrud *et al*, 2020). This transmission can be reduced through different preventive measures such as facemasks, hand hygiene, avoidance of public contact, case detection, contact tracing, and quarantines (Adhikari *et al*, 2020)**.** The corona virus can affect all age groups, but the risk of death from the virus was primarily associated with age and chronic diseases (Liu *et al*, 2020; Zhou, et *al*, 2020).

Globally COVID-19 is responsible for 34.8 million cases and over 1 million deaths, as of the 04 October 2020 (WHO, 2020). In Africa over 1 million cases and 26,264 deaths were registered as of the 04 October 2020 (WHO, 2020)**.** It is estimated that, COVID-19 will cause 1,222.3 million infections, 22.5 million hospitalizations and 3.3 million deaths in Africa in this year in the absence of any intervention (UNECA, 2020). Estimates showed

that, COVID-19 would have resulted in 7 billion infections and 40 million deaths globally this year if no action is taken (Walker *et al*, 2020).

Beyond causing morbidity and mortality, the virus can cause a great impact on the economy of once country. The report of the United Nations (UN), has estimated that because of COVID-19 the African continent will face 1.4 percentage point of Gross Domestic Product (GDP) decline (UNECA, 2020). Generally to prevent the diverse impacts of COVID-19 it is very essential to focus on prevention measures, hence the virus has no medication or vaccine. Facemasks combined with other prevention measures are very essential for preventing the transmission of COVID-19.

Despite, the recommendations on facemask wearing is controversial across countries, since the outbreak of COVID-19, the use of facemasks has become increased in China and other Asian countries such as South Korea and Japan (Feng *et al*, 2020) since the virus can transmitted through respiratory droplets even from asymptomatic individuals (He *et al*, 2020; Ganyani *et al*, 2020). In USA the number of COVID-19 cases, hospitalizations, and deaths were reduced dramatically following facemask wearing **(**Lyu and Wehby, 2020; Zhang *et al*, 2020). Even if, facemask is a potential tool to tackle the COVID-19 pandemic, many people's including taxi drivers not regularly using the facemask due to different unknown reasons.

In Ethiopia, the first case of COVID-19 was reported on 13 March 2020, since then the number of cases is increasing from time to time following improved testing. WHO reported that in the country there are 76,988 confirmed cases and 1,207 deaths recorded as of the 04 October 2020 (WHO, 2020)**.** This number indicated that different intervention strategies are needed for the prevention of its spread. In response to this, the government of Ethiopia imposes restriction to the movement of taxi in Ethiopia including Dessie City and Kombolcha towns. This restriction, made harder for local taxi drivers who rely on taxi driving. Then the

government allowed the movement of taxi by giving special attention for prevention measures, such as reducing the number of travellers by half and also recommends hand sanitizer and facemask use (Ahmed, 2020; Debela, 2020)**.** Currently the government of Ethiopia imposes the use of facemask for all people of the country as a means of reducing the transmission. These all showed the importance of facemask as a prevention measures for COVID-19.

Despite these efforts, the virus is increasing from time to time at alarming rate; therefore it is important to strengthen the prevention measures. Assessing taxi drivers COVID-19 prevention measures plays a great role in the prevention of the virus, as they had daily close contact with the community during providing transportation service. The result of the study in six Asian countries revealed that from 103 work-associated COVID-19 cases, 18% happened among drivers and transport workers **(**Lan *et al*, 2020). Even if taxi drivers are more vulnerable to the virus, there is no study conducted in Dessie City and Kombolcha Towns with regard to proportion of facemask wearing and associated factors among taxi drivers. Therefore, this study aimed to asses' the proportion of facemask wearing and associated factors among taxi drivers in Dessie City and Kombolcha Towns, Northeastern Ethiopi[illegible]

Materials and Methods

Study Design and Study Area

A cross-sectional study was conducted from July to August, 2020 in Dessie City and Kombolcha Town in North-eastern Ethiopia. Dessie City is found in South Wollo Zone, Eastern margin of Amhara regional state and in North central part of Ethiopia at a distance of 401km from Addis Ababa. The town lies on the intersection of 11°8′N 39°38′E. According to the 2007 population and housing census projection, Dessie District had a total population of 212,436 in 2014. Of the total, 83.6% (177,688) lived in urban *kebeles and* 34,748 (16.4%)

in peri-urban kebeles [1]. There are 613 and 70 taxis in Dessie City and Kombolcha Towns, respectively.

Study Population, Inclusion and Exclusion Criteria

All taxi drivers in Dessie City and Kombolcha Towns were the source populations of the study. The study population were all randomly selected taxi drivers. From the study population, temporary taxi drivers were excluded. Taxi drivers who are permanent in the selected fermata at the time of data collection were included.

Sample Size Determination and Sampling Procedure

Sample size was determined using single population proportion ($n = \frac{(z_{a/2})^2 * p(1-p)}{d^2}$) formula by considering the assumptions of proportion of facemask wearing in Dessie City and Kombolcha Town was 50% since there was no study that has been conducted in the study area so far, 95% confidence level, and a 5 % margin of error (*d*). After considering 10% non-response rate from the initial calculated sample size, the final sample size becomes 422.

From a total of eight taxi fermatas found in Dessie City, five of them were selected randomly. In addition, in Kombolcha town there are five taxi fermatas, four of them were selected randomly. Lastly, proportional allocation technique was used to determine the number of taxi drivers which have to be included from each fermata. Then, simple random sampling techniqu[illegible]as employed to select taxi drivers from the respective ~~fermatas~~.

Outcome variable measurement ~~and estimation of proportion of facemask wearing~~

The study outcome variable was wearing or not wearing of facemask. Taxi drivers wearing the facemask were coded ~~by~~ 1 ~~or Taxi drivers who do not wear the facemask was coded 0.~~

Based on the facemask use, the proportion of facemask wearing was estimated by dividing the number of facemask users to the total study participants in Dessie City and Kombolcha Towns.

Operational definitions

Facemask wearing- Using a loose-fitting, disposable or reusable device that creates a physical barrier between the mouth and nose of the wearer and potential contaminants in the immediate environment.

Good knowledge- Taxi drivers who answer above and equal to the mean of knowledge questions out of 11 knowledge questions.

Poor knowledge- Taxi drivers who answers below the mean of knowledge questions out of 11 knowledge questions.

Good attitude- Taxi drivers who answer above and equal to the mean of attitude questions out of 15 attitude questions.

Poor attitude - Taxi drivers who answers below the mean of attitude questions out of 15 attitude questions.

Proportion of facemask wearing- Taxi drivers who wear the facemask at the time of the data collection by covering the nose, mouth, lower jaw and facial hair and divided by the total number of study participant.

Data Collection and Quality Assurance

A pretested structured questionnaire adapted from published articles, WHO, Ethiopian Ministry of Health (EMOH), and Ethiopian Public Health Institute (EPHI) was used to assess facemask wearing practice among taxi drivers. The questionnaire was prepared in English and translated to Amharic and then re-translated back to English. The questionnaire has three parts; Part I includes questions about Socio- demographic and economic factors; Part II about knowledge and attitude related factors and Part III about behavioural factors. Two data

collectors and one supervisor were recruited for data collection. The data was collected by face to face interview at the working site and by observing utilization of facemask by keeping a minimum of 1m distance from the interviewees. In addition, the data collectors were used a facemask and a hand sanitizer throughout the data collection process.

To ensure the quality of the data, two day training was given for 2 data collectors and 1 supervisor about the objective of the study, data collection tools, ethical issues and other consideration. The data collector was also trained about COVID-19 prevention measures. In addition before the start of the actual data collection, the questionnaire was pretested on 5% of the sample size from out of the selected fermatas of both Dessie City band Kombolcha Towns to check the clarity and consistency of the questionnaire. The questionnaire was checked daily by the principal investigator and supervisor for completeness and consistency.

To ensure the validity of the survey tool, the questionnaire was developed after reviewing published articles. To check the reliability of the information entered, 10% of the study participants was randomly selected and re interviewed by another interviewer. The accuracy of data entries was also checked by randomly selected 10% of the questionnaires.

Data Management and Statistical Analysis

The collected data was checked, coded and entered into EpiData version 3.4 and exported to SPSS version 25.0 for data cleaning and analysis. Descriptive statistic such as frequencies and percentages were calculated for categorical variables and mean with standard deviations for continues variables to examine the overall distribution.

Data was analysed using binary logistic regression model with 95% confidence interval (CI) and Bivariable (Crude Odds Ratio [COR]) and multivariable (Adjusted odds ratio [AOR]) analysis were employed. From the Bivariable analysis, variables with $p<0.25$ was considered

for multivariable analysis. From the multivariable logistic regression analysis, variables with a significance level at $p<0.05$ was taken as statistically significant and independently associated with facemask wearing among taxi drivers. Multicollinearity was checked among independent variables using standard error and model fitness was also checked using the Hosmer Lemeshow test with *p*-value >0.05.

Ethics approval and consent to participate

Ethical clearance was obtained from the ethical review committee of Wollo University College of Medicine and Health Sciences. Before each interview, data collectors explained the purpose of the study, gave assurance of confidentiality, addressed any other ethical issues and obtained informed verbal consent from each taxi drivers.

Results

Socio-demographic and economic characteristics of the study participants

Out of 422 taxi drivers to participate in the study, 417 were participated with a response rate of 98%. From the total 417 participants the majority 224(53.7%) of the taxi drivers were aged between 18 and 29 years and some 66(15.8%) were ≥40 years and the mean age was 29.61 (±7.264 SD [standard deviations]). Regarding to educational level, the majority 181(43.4%) of the driver's education status were secondary level and some 66(15.8%) were college and above (Table 1).

Knowledge and Attitude related factors

Out of the total 417 taxi drivers the majority 291(69.8%) of them had a good knowledge about Covid-19 were as some 126(30.2%) had poor knowledge. With regard to attitude about

the disease the majority 282(67.6%) had good attitude and some 135(32.4%) had poor attitude towards COVID-19 (Table 2).

Behavioural related factors

Among 417 taxi drivers nearly half 204(48.9%) of the drivers feel as they are vulnerable to the virus while some 213(51.10%) did not feel vulnerability. The majority 262(62.8) of taxi drivers fears the virus and some 155(37.2%) did not fears the virus (Table 3).

Proportion of Facemask Wearing

The proportion of facemask wearing among taxi drivers was 54.68% [95%CI: 50.1 -59.7]. The majority 243(58.3%) of the drivers were using cloth facemasks, followed by N95 facemasks 102(24.5%) and surgical facemasks 72(17.3%). With regard to the disposal of facemask, the majority 217(52.0%) of the drivers dispose the facemask in a pit and some 7(1.7%) practice open disposal (Fig 1).

Fig 1 Proportion of Facemask wearing among taxi drivers in Dessie ~~City~~ and Kombolcha Town, Northeast Ethiopia, June to August 2020

Factors Associated with Facemask Wearing

From multivariable logistic regression analysi[illegible]arital status, fear of the disease, believing in the effectiveness of facemask and government pressure showed significant association with the facemask wearing among taxi drivers.

Married individuals were 3.14 times more likely to wear facemask than unmarried individuals (AOR=3.14, 95%CI: 1.97-5.01). Fear of the disease also showed significant association with facemask wearing. Individuals who fear the virus were 2.1 times more likely to wear facemasks than individuals who do not fear the disease (AOR=2.1, 95%CI: 1.28-3.47). On the other hand individuals who believe in the effectiveness of facemask were 5.6 times more likely to wear facemask than those who do not believe in the effectiveness of

facemask (AOR=5.6, 95%CI: 3.1-10.16). Similarly drivers who report the presence of government pressure were 3.6 times more likely to wear facemask (AOR=3.6, 95%CI: 2.16-6.13) (Table 4).

Discussion

This Cross-sectional study was conducted with the aim of determining the proportion and associated factors of facemask wearing among taxi drivers in urban areas of Dessie and Kombolcha. We found that the proportion of facemask wearing among taxi drivers was 54.7% and facemask wearing among taxi drivers, was significantly associated with marital status, fear of the corona disease, a belief on the effectiveness of facemask wearing and government pressure.

The finding of this study showed that the proportion of facemask wearing among taxi drivers was 54.68%. This finding is in line with the study which was conducted in china among elementary school student which is 51.60% (Chen *et al*, 2020). Lower than the proportion in South Korea which is 63.2% (Lee and You, 2020) in Beijing 70% (Yang *et al*, 2020), and in Hong Kong 61.2% **(**Tang and Wong, 2004) but higher than in USA 41% (Haischer *et al*, 2020), Hong Kong, 21 % (Lau *et al*, 2010), Japan, 38% (Wada, Oka-Ezoe and Smith, 2015) and in Korea 15.5% (Jang *et al*, 2019). The higher proportion of facemask wearing in this study compared to those other areas might have been due to the pressure from the government to wear the facemask. In addition to this the difference can be due to different study area and study period.

Our study revealed that the proportion of facemask wearing was 3.14 times higher among married taxi drivers compared with unmarried taxi drivers. This can leads the unmarried individuals to be more vulnerable to COVID-19. This finding is similar with the study which was conducted in Hong Kong **(**Tang and Wong, 2004). The reason for marital status is the

factor for the wearing of facemask might be due to the fact that married individuals feel responsible for the health of themselves and also their family than that of unmarried individuals.

This study showed that individuals who had a belief in the effectiveness of wearing facemasks were 5.6 times more likely to wear facemasks. Consistent with our finding, several studies revealed that believing in the effectiveness of facemask can play a great role in facemask wearing behaviour **(**Tang and Wong**,** 2004)**, (**Lau *et al.*, 2008)**, (**Lau *et al.*, 2010**)** ,and (Ferng *et al.*, 2011). This can be due to human behaviour of doing what they belief. Even if lockdown and self-isolation plays a major role causing 66% reduction of cases and 66% increase of recovered cases **(**Chintalapudi *et al.*, 2020), in places where difficult to keep social distance wearing facemask can be effective method to prevent the spread of the disease. The result of a recent study also revealed that facemask is effective against respiratory infections **(**Barasheed *et a*l, 2020)**.**

Concerning to fear of the corona disease, this study showed that the proportion of facemask wearing was higher among taxi drivers who fears corona disease. Individuals who fear the disease were 2.1 times more likely to wear facemasks than those who do not fear the disease. This finding is similar with the study which was conducted in Hong Kong (Tang and Wong**,** 2004). The finding is also consistent with the recent study which was conducted among Turkish adults during COVID-19 outbreak **(**Yildirim, Geçer, and Akgul, 2020). The possible reason is that, the serious nature of the epidemic made the drivers to fear the disease and this leads to the wearing of facemasks. Although facemask wearing is a protective measure, the result of this study showed that it is more practiced among taxi drivers who fear the corona disease.

This study also found out that, drivers who reported that there is pressure from the government to wear facemask were 3.6 times more likely to wear facemasks than others. This result is supported by a recent study in USA that facemask wearing showed dramatic increase from 41% to 90% following government pressure (Haischer *et al*, 2020). This finding is also similar with the studies which were conducted in Hong Kong (Tang and Wong**,** 2004), in Japan (Burgess and Horii, 2012) and in Mexico City public transportation during the influenza outbreak (Condon and Sinha, 2010). The reason is that in Ethiopia facemask wearing is a mandatory for the taxi drivers and they will pay 1000 Ethiopian birrs if they do not wear the facemask while driving. Even if, facemask wearing is an obligation which is imposed to taxi drivers it is mostly practiced in places where there is a regular and strong control by the traffic polices.

The study has some limitations: one of the limitations of the study was that the study did not investigate household, individual and community level factors using multilevel analysis and there was scarcity of literatures on the proportion of facemask wearing among taxi drivers; thus, the discussion was made on the basis of findings with other areas. Despite these limitations, the study provides new insight into the extent of facemask wearing among taxi drivers in Dessie City and Kombolcha Towns, Ethiopia.

Conclusion

This study identified socio-demographic and economic factors, knowledge and attitude related factors and behavioural factors associated with facemask wearing among taxi drivers in Dessie City and Kombolcha Towns. The result of this study revealed that the proportion of facemask wearing among taxi drivers was 54.68%. This result indicates that the proportion of facemask wearing is relatively low in Dessie City and Kombolcha Towns. The main factors that significantly associated with facemask wearing were marital status, fear of

the disease, belief in the effectiveness of facemask and government pressure**.** Dessie City and Kombolcha Towns Health offices should have to motivate health extension workers to give health education for the taxi drivers on proper utilization of facemasks**.** It is also strongly recommended that Dessie City and Kombolcha towns traffic polices must have to control the proper and regular wear of facemasks by taxi drivers.

Acknowledgements

We would also like to extend our special thanks to data collectors, supervisor, and the study participants for their valuable contribution for the study.

Abbreviations

AOR, Adjusted Odds Ratio; COR, Crude Odds Ratio, CI, Confidence Interval

References

AHMED, A.2020. FEDERAL NEGARIT GAZETTE OF THE FEDERAL DEMOCRATIC REPUBLIC OF ETHIOPIA. No. 35, 20th April

Anfinrud, P., Bax, C.E., Stadnytskyi, V., and Bax, A. 2020. Could SARS-CoV-2 be transmitted via speech droplets?

Barasheed, O., Alfelali, M., Mushta, S., Bokhary, H., Alshehri, J., Attar, A., Booy,R., Rashid,H.2016. Uptake and effectiveness of facemask against respiratory infections at mass gatherings: a systematic review

Burgess A, Horii M. Risk, ritual and health responsibilisation: Japan's 'safety blanket' of surgical face mask-wearing. Sociol Health Illn 2012; 34:1184-98.

Chan, JF, Yuan, S, Kok, KH, To, KK, Chu, H, Yang, J, Xing, F, Liu, J, Yip, CC, Poon, RW, et al. 2020. A familial cluster of pneumonia associated with the 2019 novel coronavirus indicating person-to-person transmission: a study of a family cluster. Lancet. 395(10223):514–523.

Chen, X., Ran, L., Liu, Q., Hu, Q., Du, X., and Tan, X.(2020). Hand Hygiene, Mask-Wearing Behaviours and Its Associated Factors during the COVID-19 Epidemic: A Cross-Sectional Study among Primary School Students in Wuhan, China

Chintalapudi, N., Battineni, G., Amenta, F. 2020. COVID-19 virus outbreak forecasting of registered and recovered cases after sixty day lockdown in Italy: A data driven model approach. 53, 396-403

Condon, B. J., and Sinha, T. (2009). Who is that masked person: The use of face masks on Mexico City public transportation during the Influenza A (H1N1) outbreak

Debela, B., K. 2020. The COVID-19 Pandemic and the Ethiopian Public Administration: Responses and Challenges

Feng, F., Shen, C., Xia, N., Song,w., Fan,M., Cowling,B.*(2020).* Rational use of face masks in the COVID-19 pandemic. pp.1–9, doi.org/10.1016/S2213-2600 (20)30134-X

Ferng YH, Wong-McLoughlin J, Barrett A, Currie L, Larson E.92011). Barriers to mask wearing for influenza-like illnesses among urban Hispanic households. Public Health Nurs 2011; 28:13-2

Ganyani T, Kremer C, Chen D, Torneri A, Faes C, Wallinga J, et al. Estimating the generation interval for coronavirus disease (COVID-19) based on symptom onset data, March 2020. Euro Surveill. 2020; 25(17): 2000257.

Guan, W., Ni, Z., Hu, Y., *et al.2020.* Clinical characteristics of 2019 novel coronavirus infection in China, medRxiv. doi: https://doi.org/10.1101/2020.02.06.20020974

HaischerI, M.H., Beilfu,R., Hart,M.R., Opielinski,L., Wrucke, D., Zirgaitis,G., Uhrich, T.D., and Hunter,S.K. 2020.**I2**Who is wearing a mask? Gender-, age-, and location-related differences during the COVID-19 pandemic. https://doi.org/10.1371/journal.pone.0240785

He X, Lau EHY, Wu P, Deng W, Wang J, Hao X, et al. Temporal dynamics in viral shedding and transmissibility of COVID-19. Nat Med. 2020; 26(5): 672–675. https://doi.org/10.1038/s41591-020-0869-5

Holshue, M. L., Debolt, C., Lindquist, S., Lofy, K.H, Wiesman, J., and Bruce, H., 2020. First Case of 2019 Novel Coronavirus in the United States. pp.1–8DOI: 10.1056/NEJMoa2001191

Huang,C., Wang, Y., Li, X., Ren, L.,Zhao,J., Hu,Y., Zhang,L., Fan,G., Xu,J., Gu,X., Cheng, Z., Yu, T., Xia,J., Wei,Y., Wu,W., Xie, X., Yin,W., Li,H., Liu, M.,Xiao, Y.,Gao,H., Guo,L., Xie,J.,Wang,G., Jiang,R., Gao, Z.,Jin,Q., Wang, J., Cao,B.2020. Clinical features of patients infected with 2019 novel coronavirus in Wuhan, China.

Jang ,W., Cho ,S.,2,y, Jang , D.H., Kim,U. , Jung,H. , Lee,J.Y. and Eun .S.J.(2015). Preventive Behavioral Responses to the 2015 Middle East Respiratory Syndrome Coronavirus Outbreak in Korea

Jefferson, T.; Del Mar, C.B.; Dooley, L.; Ferroni, E.; A Al-Ansary, L.; A Bawazeer, G.; Van Driel, M.L.; Foxlee, R.; Rivetti, A. Physical interventions to interrupt or reduce the spread of respiratory viruses: Systematic review .*BMJ* 2009, *339*, b3675.

Lakew, Y., Reda, A. A., Tamene, H., Benedict, S. & Deribe, K. 2013. Geographical variation and factors influencing modern contraceptive use among married women in Ethiopia: evidence from a national population based survey. *Reproductive health,* 10, 52.

Lan, F., Wei1, C., Hsu, Y., Christiani1, C.D., Stefanos, N. Kales (2020).Work-related COVID-19 transmission in six Asian countries/areas: A follow-up study. 15(5): e0233588. https://doi.org/10.1371/journal.pone.0233588

Lau JT, Kim JH, Tsui HY, Griffiths S. Perceptions related to bird-to-human avian influenza, influenza vaccination, and use of face mask. Infection 2008; 36:434-43

Lau, J. T. F., Griffiths, S., Choi, K. and Lin, C. (2010). Prevalence of preventive behaviours and associated factors during early phase of the H1N1 influenza epidemic. 38:374-80.

Lee, M., and You, M. (2020). Psychological and Behavioral Responses in South Korea During the Early Stages of Coronavirus Disease 2019 (COVID-19). doi:10.3390/ijerph17092977

Li, Q., Guan, X., Wu, P.,Wang, X., Zhou, L., Tong, Y., Ren, R., … (2020). Early Transmission Dynamics in Wuhan, China, of Novel Coronavirus–Infected Pneumonia: DOI: 10.1056/NEJMoa2001316

Liu, K.., Fang, Y., Deng, Y., Liu, W., Wang, M., Ma, J., Xiao, W., Wang, Y., Zhong, M., LiC.,, Li,G., Liu,H.2020. Clinical characteristics of novel coronavirus cases in tertiary hospitals in Hubei Province**.** 133(9)

Lyu, W and Wehby,G ,L. 2010. Community Use of Face Masks and COVID-19: Evidence from a Natural Experiment of State Mandates in the US. doi: 10.1377/hlthaff.2020.00818

Phelan, A.L., Katz, L. Gostin, L. O. 2020 The Novel Coronavirus Originating in Wuhan, China Challenges for Global Health Governance.

Sim, S.W., Moey,K.S., and Tan,N.,C. (2014). The use of facemasks to prevent respiratory infection: a literature review in the context of the Health Belief Model .55(3): 160-167, doi:10.11622/smedj.2014037

Tang, C., S., and Wong, C. 2004. Factors influencing the wearing of facemasks to prevent the severe acute respiratory syndrome among adult Chinese in Hong Kong.39 (2004) 1187–1193. doi:10.1016/j.ypmed.2004.04.032

Taylor, M., Raphael, B., Barr, M., Agho, K., Stevens, G. Jorm, L., 2009. Public health measures during an anticipated influenza pandemic: Factors influencing willingness to comply 2009:2 9–20

UNECA (2020). COVID-19 in Africa: Protecting Lives and Economies .pp.1–49.

Walker,P., Whittaker,C., Watson,O., Baguelin,M., Ainslie, K., Bhatia,S.,Bhatt,S., 2020. The Global Impact of COVID-19 and Strategies for Mitigation and Suppression .7(18), pp.1–19. DOI: https://doi.org/10.25561/77735.

Wang, D, Hu, B, Hu, C, Zhu, F, Liu, X, Zhang, J, Wang, B, Xiang, H, Cheng, Z, Xiong, Y, et al. 2020. Clinical characteristics of 138 hospitalized patients with 2019 novel

coronavirus–infected pneumonia in Wuhan, China. JAMA doi:10.1001/jama.2020.1585.

Wong, G., Liu, W. , Liu ,.Y., Zhou. B., Bi, Y., and Gao, G. F. (2015). MERS, SARS, and Ebola: The Role of Super-Spreaders in Infectious Disease. Doi.org/10.1016/j.chom.2015.09.013

Wada, K., Oka-Ezoe, K., and Smith, D. R. (2012). Wearing face masks in public during the influenza season may reflect other positive hygiene practices in Japan. 12:1065, 1-6.

WHO(2020). Coronavirus Disease 2019 (COVID-19): Data as received by WHO from national authorities, as of 04 October 2020.

Yang, Y., Lu, Q., Liu, M., Wang, Y, Zhang, A , Jalali, N., Dean, N. , Longini, I., Halloran M. E., Xu, B., Zhang, X-A., Wang, L.P., Liu, W., and FangL.Q.(2020) .Epidemiological and clinical features of the 2019 novel coronavirus outbreak in China.

Yildirim, M., Geçer, E., & Akgul, O. 2020. The impacts of vulnerability, perceived risk, and fear on preventive behaviours against COVID-19

Zhang, K., Vilches, T.N., Tariq, M., Galvani, A P., Moghadas, S. M.2020. The impact of mask-wearing and shelter-in-place on COVID-19 outbreaks in the United States. DOI: https://doi.org/10.1016/j.ijid.2020.10.002

Zhou,P., Zhang, D., Wang ,W., Hu,B., Zhang, L., Li, X,N., Dean,N.E., Longini,I., Halloran,M.E., Xu,B., Zhang,X.A., Wang,l.p., Liu,W.,and Liu,W and Fang,L.Q. (2020). A pneumonia outbreak associated with a new coronavirus of probable bat origin.

Zhou,F., Yu,T., Du,R., Fan,G., Liu,Y., Liu, Z., Xiang,J., Wang, Y., Song,B., Gu,X., Guan,L., Wei,Y., Li,H., Wu,X., Xu, J., Tu, S., Zhang,Y., Chen, H., Cao,B.2020.

Clinical course and risk factors for mortality of adult inpatients with COVID-19 in Wuhan, China: a retrospective cohort study

Zhu,N., Yang ,X.L. ,Wang ,X.G., Hu,B., Zhang,L., Li ,X. Yang ,B…(2020). A Novel Coronavirus from Patients with Pneumonia in China, 2019. DOI:10.1056/NEJMoa2001017.

Table 1: Taxi drivers related socio-demographic factors in Dessie city and Kombolcha town, Northeast Ethiopia, June to August 2020.

Variables	Frequency (*N* = 417)	Percentage (%)
**Drivers age (in years)**		
18-29	224	53.7
30-39	127	30.5
≥40	66	15.8
**Drivers education status**		
Primary(8)	170	40.8
Secondary (9-12)	181	43.4
College and above	66	15.8
**Marital status**		
Unmarried	178	42.7
Married	239	57.3
**Religion**		
Muslim	266	63.8
Christian	151	36.2
**Place of residence**		
Urban	407	97.6
Rural	10	2.4
**Monthly income**		
≤142.5$	270	64.7
>142.5$	147	35.3
**Household size**		
≤5	376	90.2
>5	41	9.8
**Mean driver age(in year)**	29.61((±7.264SD)	

SD= Standard deviation

Table 2: Knowledge and Attitude related factors in Dessie city and Kombolcha town, Northeast Ethiopia, June to August 2020.

**Variables**	**Frequency (*N* = 417)**	**Percentage (%)**
**Knowledge**		
Good	291	69.8
Poor	126	30.2
**Attitude**		
Good	282	67.6
Poor	135	32.4

Table 2: Behavioural related factors in Dessie city and Kombolcha town, Northeast Ethiopia, June to August 2020

Variables	Frequency (N = 417)	Percentage (%)
**Feeling of vulnerable to COVID-19**		
Yes	204	48.9
No	213	51.1
**Knowing infected individuals with COVID-19**		
Yes	27	6.5
No	390	93.5
**Fear of COVID-19**		
Yes	262	62.8
No	155	37.2
**Worry that Dessie city/Kombolcha town would become a quarantine city/town**		
Yes	165	39.6
No	252	60.4
**Believing that wearing facemask could prevent contracting and spreading of COVID-19**		
Yes	309	74.1
No	108	25.9
**Difficulty in obtaining facemask**		
Yes	133	31.9
No	284	68.1
**Discomfort when wearing a facemask**		
Yes	219	52.5
No	198	47.5
**Local government pressure**		
Yes	301	72.2
No	116	27.8
**Family members' encouragement**		

Yes	210	50.4
No	207	49.6
**Passengers encouragement**		
Yes	160	38.4
No	257	61.6
**Work place acceptance of wearing facemask**		
Yes	263	63.1
No	154	36.9
**Knowing that corona is a fatal disease**		
Yes	385	92.3
No	32	7.7

Table 3: Multivariable analysis of Facemask wearing among taxi drivers in Dessie city and Kombolcha town, Northeast Ethiopia, June to August 2020

Variables	Facemask wearing		AOR(95%CI)	*P-value*
	Yes	No		
	n(%)	n(%)		
**Marital status**				
Married	156(37.4)	83(19.9)	3.14 (1.97-5.01)	< 0.001
Unmarried	72(17.3)	106(25.4)	1	
**Fear of the disease**				
Yes	173(41.5)	89(21.3)	2.12 (1.28-3.47)	0.003
No	55(13.2)	100(24)	1	
**Belief in the effectiveness of facemask-wearing**				
Yes	206(49.4)	103(24.7)	5.6 (3.1-10.16)	< 0.001
No	22(5.3)	86(20.6)	1	
**Government pressure**				
Yes	241(57.8)	68(16.3)	3.64(2.16-6.13)	< 0.001
No	60(14.4)	48(11.5)	1	

1, reference category

Click here to access/download;Figure;Figure 1.rtf

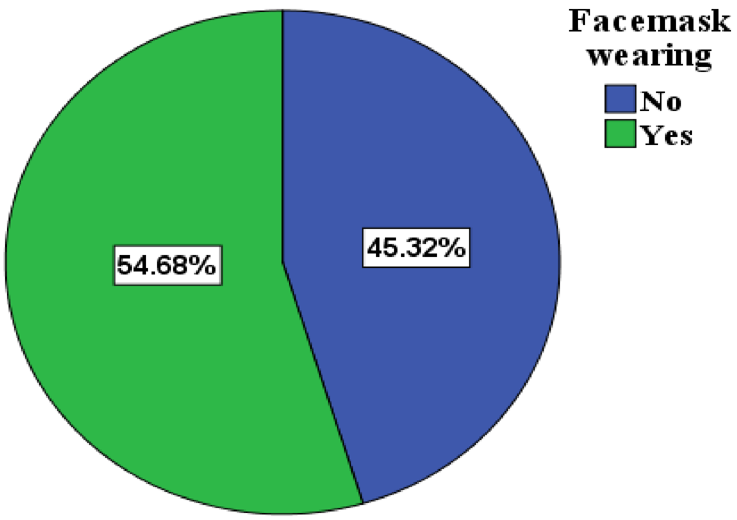

Supplement: Attachment — Submitted filename: Wearing Facemask reviewer.pdf [file pone.0247954.s005.pdf]
