# Supplementary material for: Facemask wearing to prevent COVID-19 transmission and associated factors among taxi drivers in Dessie City and Kombolcha Town, Ethiopia
Source: PLoS One. 2021 Mar 12;16(3):e0247954. doi: 10.1371/journal.pone.0247954 (PMC7954338; doi:10.1371/journal.pone.0247954)
Supplement: Attachment — Submitted filename: PLoS one Review report on facemask.docx [file pone.0247954.s004.docx]

**Review report (Dr. Habtamu Tolera)**

The manuscript reports the findings regarding “Proportion of facemask wearing to prevent COVID-19 and associated factors among taxi drivers in urban areas of Dessie

City and Kombolcha Towns in Ethiopia”. This kind of research topic is much relevant, timely and worrying much in the context of LMICs like Ethiopia and the pandemic today’s world as a whole. The analysis was well done. However, I have some concerns (on heading, abstract, methodology, and result sections) which needed be addressed to get published in PLoS ONE. Author(s) can also refer to the attached pdf file.

- Authors did not give line pages to make review work ease. Why??

**Heading of the study:**

- Authors need to rewrite the word “Facemask” as two words (face mark), not as one word as author(s) described it in the title). keep the consistency across the manuscript.
- Authors are calling Dessie as a city. I suggest they need to use both Dessie and Komblcha as a town because both are really towns. In the current stastus, Dessie and her sister other urban centers like Debre markos, Nekemte, etc do not qualify for the standard of to be named as city (such hierarchy). Even regional capitals do not officially maintain such a rank except Addis.

**Abstract**

- The last sentence of the “Background” section under the Abstract is too long sentence and lacks language clarity. It has been rewritten.
- The “Conclusion” section under the Abstract which was submitted online in the submission system (first page of the submission system) was not complete. Authors need to recheck.

**Introduction:**

- PLoS ONe use Vancouver styles of citations, use series Arabic numerals or number citations inside a rectangular shaped bracket, [ ]. For further insight, get and read in-text-citation styles and bibliography writing from authors guidelines of the Journal. So, they need to rework accordingly.
- *Authors try to cite or use international and national figures (reports) in the Introduction section to describe the background picture of the study topic. In this regard it is well done. However, I have some concerns.The introduction part needs more detail review of relevant* ***empirical works the world experienced*** *on pandemic diseases like COVID-19 in different time. I suggest that authors improve the description in this regard to provide more justification for their study, specifically, they should expand upon the knowledge gap being filled citing adequate related works.*

**Methodology:**

- Under study design and study areas, Why opt for Dessie and Kombolcha towns than other higher urban center in the region? Here, author(s) needs to justify their rationale. In the same section, authors stated something contradicting with what they reported in the " Abstract" section under the "Method". There in the Abstract section and in each Table caption, they mentioned that the study was conducted June to August 2020 whereas here they stated it was conducted from July to August. Keep the consistency across the manuscript. Authors also said like this, ***the town lies on the intersection of 11°8′N 39°38′E.*** this shows a point, not an area. I hope, Dessie is a polygon made up of two or more kebeles. They need to use both the latitudinal and longitudinal extents of the two urban centers independently to depict their astronomical location because these towns are here polygons (areas) since they are study settings, should not be a point in such a study. I also advise authors to show the location of the study areas mapping the two study settings using GIS environment.
- Under Methodological part, there is a sub-section called **“**Outcome variable measurement and ***estimation of proportion of facemask wearing*”** I have found this miss placed sub heading, no need of stating it here as a method of data analysis because it is expected (even you described it in your method of Data magt and analysis sub-section) that authors undertake descriptive statistics like proportion, cross tabs, and like that below. avoid such repetition of data analysis from here. So, advise author(s) move it (this sub-section) immediate to the "Result" section and reportedly presents the two prevalence, wearing and not wearing face cover. authors also gave definition for Proportion of facemask wearing under Operational definitions below. Likewise edit the coding of outcome variables like this, **…**  *otherwise, those who do not wear the face mask was coded 0.*

**Ethics approval and consent to participate**

- Cite a protocol code that verified approval IRB of the WU/college

**In text citation**

- PLoS ONe use Vancouver styles of citations, use series Arabic numerals or number citations inside a rectangular shaped bracket. read in-text-citation and bibliography citation styles of authors guideline. So, rework accordingly

.

End
