# Supplementary material for: Facemask wearing to prevent COVID-19 transmission and associated factors among taxi drivers in Dessie City and Kombolcha Town, Ethiopia
Source: PLoS One. 2021 Mar 12;16(3):e0247954. doi: 10.1371/journal.pone.0247954 (PMC7954338; doi:10.1371/journal.pone.0247954)
Supplement: Attachment — Submitted filename: Response to reviwers.docx [file pone.0247954.s007.docx]

Dear Editor

Thank you for your letter with a decision of **minor revision** needed. We agree with all the comments/questions raised and please find for each below.

Response to the editor comment:

The manuscript has greatly improved from the original one. However, English language still need to be paid attention to. I recommend that you seek the help from someone offering professional English editing services.

**Response: We appreciate your concern and we received English language editing support from the native English speaker. We acknowledged the expert in page 16 in line 376 to 377 of the acknowledgment section. I marked areas within the red color that language improvement done throughout the manuscript. Please see also the areas that language improvement done. See the red marked section of the revised manuscript.**

In addition, the titles of tables and Figures should be revised. Table 1 title should read " Bivariate analysis of socio-demographic and economic factors associated with facemask wearing among .........."/ Table 2 title should read " Bivariate analysis of behavioral related factors associated with face mask wearing among  .....". Table 3 should also re-written in the same way. Table 4 title should read " Multivariable analysis of factors associated with ............".

**Response: We accepted all your suggestions for the titles. Please see the titles of Table 1, 2, 3 and 4. Thank you.**

Please do the same correction to the Figure.

Response: The title of Figure 1 is ok as it is (Please see title of Figure 1).

In lines 46 to 53, you don't need include the frequency of people who posses a given property (e.g. those who wore masks). Just presenting the their percentage (relative frequency)  is enough as the frequency can be calculated from the total when the percentage is given. This will also make it easy to read these sentences.

**Response: Yes, very crucial comment. We updated the manuscript as per your suggestion please see the revised version in the abstract in page 3 line 47 to 54. See also in the result section in pages from 11 to 12 marked with red color.**

I hope that the revised manuscript is accepted for publication in PLoS ONE.

Sincerely yours,

Metadel Adane (PhD)

Wollo University, Department of Environmental Health
